# Notch signaling pathway regulates CD4^+^CD25^+^CD127^dim/−^ regulatory T cells and T helper 17 cells function in gastric cancer patients

**DOI:** 10.1042/BSR20182044

**Published:** 2019-05-14

**Authors:** Lu Yang, Ke-Lei Zhao, Lei Qin, Dan-Xia Ji, Bin Zhang, Peng-Fei Zheng, Yong-Mei Qin

**Affiliations:** 1 Department of Gastroenterology, The First Affiliated Hospital of Xinxiang Medical University, Weihui, Xinxiang, Henan Province, China; 2 Department of Oncology, The First Affiliated Hospital of Xinxiang Medical University, Weihui, Xinxiang, Henan Province, China; 3 Department of Statistics Room of Information, The First Affiliated Hospital of Xinxiang Medical University, Weihui, Xinxiang, Henan Province, China; 4 Department of Clinical Laboratory, The First Affiliated Hospital of Xinxiang Medical University, Weihui, Xinxiang, Henan Province, China; 5 Department of Thyroid Breast and Vascular Surgery, The First Affiliated Hospital of Xinxiang Medical University, Weihui, Xinxiang, Henan Province, China

**Keywords:** gastric cancer, Notch signaling, regulatory T cells, T helper 17 cells

## Abstract

Regulatory T cells (Tregs) and T helper 17 (Th17) cells contribute to cancer progression and prognosis. However, regulatory factors associated with Tregs–Th17 balance were not completely understood. We previously demonstrated an immune-modulatory capacity by Notch signaling inactivation to reverse Tregs–Th17 disequilibrium in chronic hepatitis C. Thus, the aim of current study was to assess the role of Notch signaling in modulation Tregs and Th17 cells function in gastric cancer (GC) patients. A total of 51 GC patients and 18 normal controls (NCs) were enrolled. Notch1 and Notch2 mRNA expressions were semiquantified by real-time polymerase chain reaction. Tregs/Th17 percentages, transcriptional factors, and cytokines production were investigated in response to the stimulation of Notch signaling inhibitor DAPT. Both Notch1 and Notch2 mRNA expressions were elevated in GC tissues and peripheral bloods in GC patients. CD4^+^CD25^+^CD127^dim/−^ Tregs and Th17 cells percentage was also elevated in GC patients compared with in NCs. DAPT treatment did not affect frequency of either circulating Tregs or Th17 cells, however, reduced FoxP3/RORγt mRNA expression and interleukin (IL)-35/IL-17 production in purified CD4^+^ T cells from GC patients. Moreover, blockade of Notch signaling also inhibited the suppressive function of purified CD4^+^CD25^+^CD127^dim/−^ Tregs from GC patients, which presented as elevation of cellular proliferation and IL-35 secretion. The current data further provided mechanism underlying Tregs–Th17 balance in GC patients. The link between Notch signaling and Th cells might lead to a new therapeutic target for GC patients.

## Introduction

Gastric cancer (GC) is regarded as the fourth most common cancer in men and the fifth most common cancer in women worldwide, leading to be the third cause of cancer-related deaths globally [1]. Although the advances in diagnosis and declining rate of GC, there were still more than 500000 GC-caused death every year with approximate 20% in 5-year survival rate [2]. In China, there are 29.9 new diagnosed GC per 100000 people every year, and an estimated 221478 GC-related death happens annually, which accounts for nearly half of the GC deaths all over the world [3]. More importantly, mechanism of GC pathogenesis is still not fully elucidated and relies on multiple different factors, e.g. environmental and genetic characteristics [4]. It is well accepted that exhaustion of T cells as well as dysregulation of genes and development pathways play essential role during gastric carcinogenesis [4,5]. T-bet and T helper (Th) 1-related factors are critical in tumor development regulation, and decreased T-bet expression in GC patients may be associated with pathological event leading to Th1/Th2 imbalance [6]. Moreover, the paradigm of Th1/Th2 has been challenged by identification of two distinct Th subsets, CD4^+^CD25^+^CD127^dim/−^ regulatory T cells (Tregs) and T helper 17 (Th17) cells.

Naïve CD4^+^ T cells differentiate into different Th cell lineages via activation of various transcriptional factors and induction of different cytokines. CD4^+^CD25^+^CD127^dim/−^ Tregs express high level of transcriptional factor FoxP3 and secrete interleukin (IL)-35/IL-10, and suppress activation and expansion of tumor-antigen-specific effector T cells to maintain peripheral tolerance in malignant cancers [7,8]. GC cells could induce the differentiation of Tregs [9], and elevated level of Tregs in peripheral bloods [9], tumor microenvironment [10,11], and metastatic lymph nodes [12] mediated immune suppression and related to poor prognosis of GC. Th17 cells express high level of transcriptional factor retinoic acid-related orphan receptor-γt (RORγt) and produce IL-17/IL-22, and mainly contribute to inflammation. There was a significant skewing toward a Th17 phenotype in GC tissues than in peripheral bloods, which was also an independent negative prognostic indicator [13]. Accumulation of Tregs and Th17 cells in tumor microenvironment was gradually elevated in accordance with disease progression, resulting in an imbalance between Tregs and Th17 cells in GC patients [14,15]. However, the modulatory factors in regulation of Tregs and Th17 cells balance in GC were not fully elucidated.

Notch signaling is a highly conserved intracellular communication pathway, and plays as a potential regulator during T/B lineage determination [16]. Notch receptors expression was correlated with overall worst survival for all GC patients with 20-year follow-up [17,18]. Inhibition of Notch signaling by γ-secretase inhibitor I enhanced cytotoxic effect of 5-fluorouracil in GC [19]. Our previous study revealed that Notch signaling inhibitor DAPT could affect Tregs–Th17 subset balance in patients with chronic hepatitis C virus (HCV) infection by dampening the suppressive activity of Tregs and the function of Th17 cells [20]. Thus, we hypothesized that Notch signaling pathway also modulated the balance and function of Tregs and Th17 cells in GC patients. To test this possibility, we investigated Notch1/2 expression in peripheral bloods and tumor tissues, and then assessed the effect of Notch signaling inhibition on Tregs/Th17 cells function in GC patients.

## Materials and methods

### Subjects

A total of 51 patients with pathologically confirmed GC were enrolled in the present study. All patients were hospitalized in the Department of Gastroenterology/Oncology of The First Affiliated Hospital of Xinxiang Medical University between December 2016 and October 2017. Patients with autoimmune disorder, severe bacterial infection, or chronic hepatitis virus infections were excluded from the present study. No patients received surgery, chemotherapy, or radiotherapy before sampling. The diagnosis was made according to National Comprehensive Cancer Network Clinical Practice Guidelines in Oncology for Gastric Cancer Version 3.2016 [21]. The tumor–node–metastasis (TNM) stages were evaluated according to the American Joint Committee on Cancer/Union for International Cancer Control TNM classification (7th edition). For normal controls (NCs), 18 healthy individuals who matched for mean age and sex ratio were also enrolled. The clinical characteristics of all enrolled subjects were listed in Table 1. The study conformed to the ethical guideline of 1975 Declaration of Helsinki. The study protocol was approved by the Ethics Committee of The First Affiliated Hospital of Xinxiang Medical University (2016044). Written informed consent was obtained from each subject.

**Table 1:** Clinical characteristics of enrolled subjects

	Gastric cancer patients	Healthy individuals
Case (*n*)	51	18
Gender (male/female)	38/13	12/6
Age (years)	41 (26–53)	37 (29–52)
*Helicobacter pylori* infection (*n*)	44	6
Anemia (*n*)	35	1
Low platelet count (<100×10^9^/l) (*n*)	19	0
TNM stage (I/II/III/IV)	12/14/18/7	Not available
Clinical type (intestinal/diffuse)	48/3	Not available

Data were shown as median and range.

### Isolation of peripheral blood mononuclear cells and purification of CD4^+^CD25^+^CD127^dim/−^ Tregs/CD4^+^ T cells

Peripheral blood mononuclear cells (PBMCs) were isolated by density gradient centrifugation using Ficoll-Hypaque (Sigma–Aldrich, St. Louis, MO, U.S.A.), and were cryopreserved in fetal bovine serum (FBS) with 10% dimethyl sulfoxide (DMSO). CD4^+^ T cells or CD4^+^CD25^+^CD127^dim/−^ Tregs were purified using Human CD4^+^ Cells Isolation Kit (Miltenyi, Bergisch Gladbach, Germany) or CD4^+^CD25^+^CD127^dim/−^ Treg Isolation Kit II (Miltenyi) following manufacturer’s instruction. The purity of enriched cells was more than 95% by flow cytometry determination.

### Cell culture

DAPT (CalBiochem, Merck Chemicals International, Germany) was reconstituted in DMSO to a concentration of 10 mM. CD4^+^ T cells were stimulated with DAPT at a final concentration of 20 μM for 24 h in the presence of anti-CD3/CD28 (eBioscience, San Diego, CA, U.S.A.; final concentration 1 mg/ml), and cells and supernatants were harvested for further experiments. Cells with DMSO stimulation were used as controls. In certain experiments, purified CD4^+^CD25^+^CD127^dim/−^ Tregs were stimulated with DAPT or DMSO for 24 h. Cells were washed twice, and 2.5×10^4^ of Tregs were co-cultured with autologous 10^5^ of CD4^+^CD25^−^ T cells in the presence of anti-CD3/CD28 (eBioscience) for 96 h with replacement of fresh medium containing 20 U/ml of recombinant human IL-2 (Sigma–Aldrich) 48 h post mixture.

### Real-time reverse polymerase chain reaction

Total RNA was isolated from PBMCs or tumor/peritumor tissues using RNeasy minikit (Qiagen, Hilden, Germany) following manufacturer’s instruction. cDNA was then synthesized from 1 μg of total RNA using the RevertAid™ First Strand cDNA Synthesis kit (Fermentas, Burlington, ON, Canada). Real-time reverse polymerase chain reaction (RT-PCR) was conducted on the ABI 7500 Real-Time PCR System (Applied Biosystems; Thermo Fisher Scientific, Waltham, MA, U.S.A.) using Platinum SYBR Green Master Mix (Invitrogen; Thermo Fisher Scientific, Inc.). Amplification was performed in 20 μl reaction mixture containing 10 μl Supermix, 0.8 μM of each primer and 0.1–0.5 μg template cDNA. The sequences of the primers was cited from our previous study [20]. Relative quantification of mRNA expression was calculated with the *ΔΔCt* method using the expression level of GAPDH as an internal control [22].

### Flow cytometry

Cells were stimulated with phorbol 12-myristate 13-acetate (50 ng/ml) and ionomycin (1 μg/ml) in the presence of monensin (10 μg/ml) for 6 h. Cells were transferred to FACS tubes, and anti-CD3-PerCP Cy5.5 (eBioscience), anti-CD4-FITC (eBioscience), anti-CD25-APC (eBioscience), and anti-CD127-PE Cy7 (eBioscience) were added for a 20-min incubation in darkness at 4°C. Cells were fixed by 100 μl of Fixation & Permealization Medium A (Invitrogen, Grand Island, NY, U.S.A.) for a 15-min incubation, and then were resuspended in 100 μl of Fixation & Permealization Medium B (Invitrogen) containing anti-IL-17A-PE (eBioscience) for a 20-min incubation in darkness at room temperature. Cells were analyzed with FACS Calibur analyzer (BD Biosciences Immunocytometry Systems, San Jose, CA, U.S.A.). Acquisitions were performed using Cell Quest Pro Software (BD Biosciences), and data were analyzed using FlowJo Version 7.6.2 (Tree Star Inc., Ashland, OR, U.S.A.).

### Cellular proliferation assay

Cellular proliferation was determined by Cell Counting Kit-8 (CCK-8; Beyotime, Wuhan, Hubei Province, China) following manufacturer’s instruction. All experiments were performed in triplicate on three independent occasions.

### Enzyme-linked immunosorbent assay

Expressions of cytokine in the supernatants were measured using commercial enzyme-linked immunosorbent assay (ELISA) kits following manufacturer’s instruction.

### Western blot

The protein expressions of Hes1 and Hes5 in PBMCs were measured as previously described [20]. Briefly, total proteins, which were extracted from DMSO or DAPT-treated PBMCs, were loaded on SDS-PAGE gel, and were electroblotted onto PVDF membrane. The membrane was soaked in 5% non-fat milk containing 0.05% Tween 20 in PBS for 2 h, and was then incubated overnight in the presence of rabbit anti-Hes1 (Abcam, Cambridge, MA, U.S.A.; 1:1000 dilution), rabbit anti-Hes5 (Abcam; 1:1000 dilution), or mouse anti-GAPDH (Abcam; 1:2000 dilution). Horseradish peroxidase-conjuated goat anti-rabbit or goat anti-mouse antibody IgG (Abcam; 1: 2000 dilution) was then added for additional 2-h incubation. Antigen–antibody complexes were observed by enhanced chemiluminescence (Western Blotting Luminol Reagent, Santa Cruz, CA, U.S.A.).

### Statistical analyses

Data were analyzed using SPSS Version 19.0 for Windows (SPSS, Chicago, IL, U.S.A.). Student’s *t* test or paired *t* test was used for comparison between groups. SNK-*q* test was used for comparison among groups. *P* value less than 0.05 was considered to indicate a significant difference.

## Results

### Notch1 and Notch2 expression was elevated in GC patients

**Figure 1:**
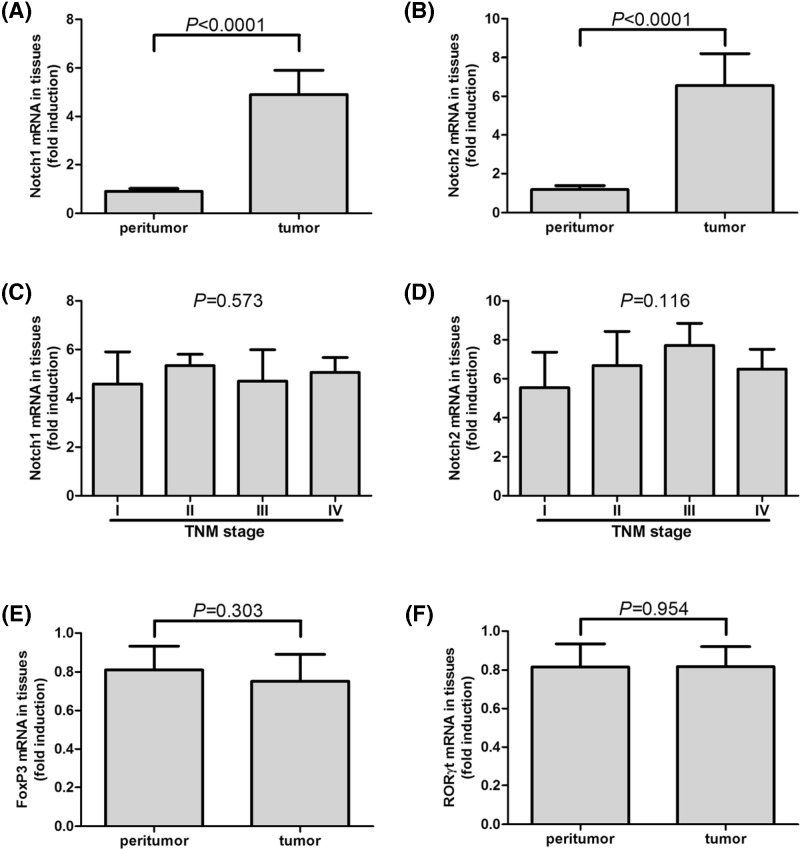
mRNA expressions of Notch1, Notch2, FoxP3, and RORγt in peritumor and tumor tissues in GC patients (*n* = 24) Both (**A**) Notch1 and (**B**) Notch2 mRNA expressions in tumor tissues was significantly elevated than in peritumor tissues. There were no remarkable differences of (**C**) Notch1 or (**D**) Notch2 mRNA expression in tumor tissues among GC patients in different TNM stages. There were no significant differences of (**E**) FoxP3 or (**F**) RORγt between tumor and peritumor tissues. Columns presented as means, and bars presented as standard deviations. Paired *t* tests or SNK-*q* tests were used for comparisons.

**Figure 2:**
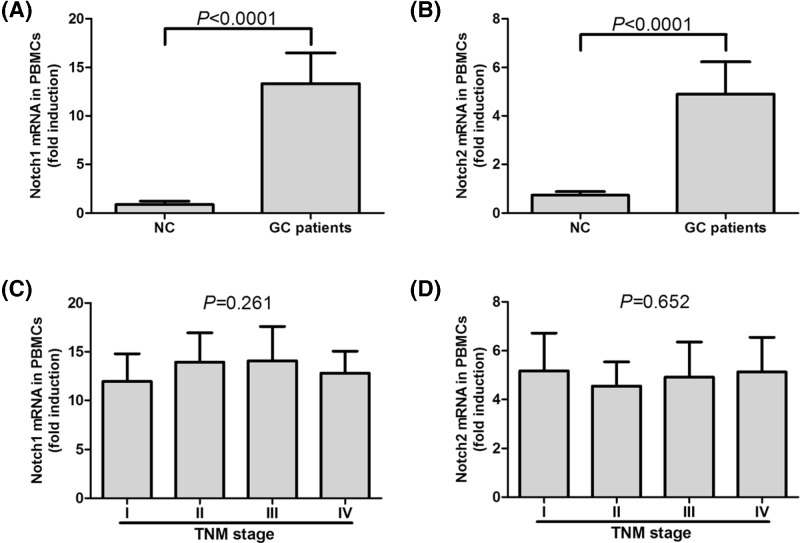
mRNA expressions of Notch1 and Notch2 in PBMCs in NC (*n* = 18) or GC patients (*n* = 51) (**A**) Notch1 and (**B**) Notch2 mRNA expressions in PBMCs was significantly elevated in GC patients in comparison with that in NC. There was no remarkable difference of (**C**) Notch1 or (**D**) Notch2 mRNA expression in PBMCs among GC patients in different TNM stages. Columns presented as means, and bars presented as standard deviations. Student’s *t* tests or SNK-*q* tests were used for comparisons.

Previous studies showed differential expression profiling of Notch1 and Notch2 in colorectal carcinoma specimens, which revealed up-regulation of Notch1 and down-regulation of Notch2 with significant relations to tumor differentiation status [23,24]. Thus, we firstly screened Notch1 and Notch2 mRNA expressions in tumor and peritumor tissues which were obtained during gastroscopic biopsy in 24 of GC patients (7 of TNM stage I, 6 of stage II, 6 of stage III, and 5 of stage IV). Notch1 and Notch2 mRNA expressions revealed approximate five-fold and six-fold elevation in comparison with in peritumor tissues, respectively (paired *t* tests, all *P*<0.0001, Figure 1A,B). However, there were no differences on tumor-resident Notch1/2 mRNA expressions among GC patients in different TNM stages (SNK-*q* tests, *P* = 0.572 and *P* = 0.116, respectively, Figure 1C,D). Moreover, mRNA relative levels corresponding to FoxP3 and RORγt were also investigated. FoxP3 and RORγt mRNA was only found to be expressed in 11 tumor tissues and seven peritumor tissues. There were no remarkable differences of FoxP3 or RORγt mRNA levels between tumor and peritumor tissues (Student’s *t* tests, *P* = 0.303 and *P* = 0.954, respectively, Figure 1E,F). mRNA expressions corresponding to Notch1 and Notch2 were also measured in PBMCs isolated from NC and GC patients. Both Notch1 and Notch2 showed significant elevation in GC patients compared with in healthy individuals, with approximate 15-fold and 5-fold increase, respectively (Student’s *t* tests, all *P*<0.0001, Figure 2A,B). There were also no differences of Notch1/2 mRNA expressions among GC patients in different TNM stages (SNK-*q* tests, *P* = 0.261 and *P* = 0.652, respectively, Figure 2C,D).

### Circulating CD4^+^CD25^+^CD127^dim/−^ Tregs and Th17 cells was elevated in GC patients

**Figure 3:**
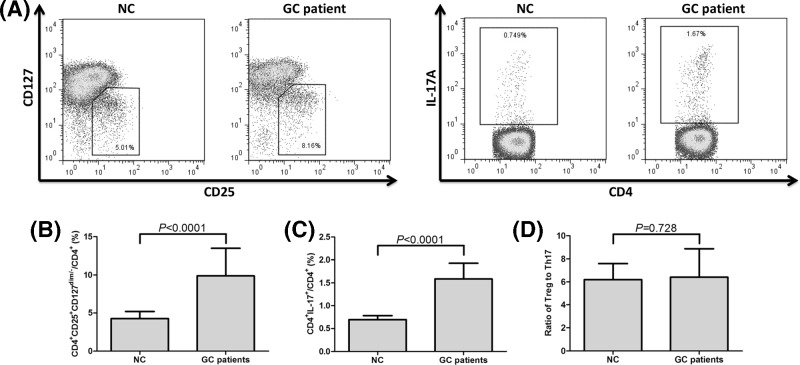
CD4^+^CD25^+^CD127^dim/−^ Tregs and Th17 cells in NC (*n* = 18) or GC patients (*n* = 51) (**A**) Representative dot plots of CD4^+^CD25^+^CD127^dim/−^ Tregs and Th17 cells in NC and GC patients. The percentage of (**B**) CD4^+^CD25^+^CD127^dim/−^ Tregs and (**C**) Th17 cells within CD4^+^ T cells was significantly elevated in GC patients in comparison with NC. (**D**) There was no remarkable difference in the ratio of Tregs to Th17 cells between NC and GC patients. Columns presented as means, and bars presented as standard deviations. Student’s *t* tests were used for comparisons.

Circulating CD4^+^CD25^+^CD127^dim/−^ Tregs and Th17 cells in all enrolled NC and GC patients were measured by flow cytometry, and the representative flow dots are shown in Figure 3A. Percentage of CD4^+^CD25^+^CD127^dim/−^ Tregs within CD4^+^ T cells was significantly elevated in GC patients when compared with NC (9.89±3.59% vs. 4.26±0.92%; Student’s *t* test, *P*<0.0001, Figure 3B). Th17 cells frequency within CD4^+^ T cells was also notably increased in GC patients in comparison with NC (1.59±0.34% *vs.* 0.70±0.09%; Student’s *t* test, *P*<0.0001, Figure 3C). To link Tregs with Th17 cells, we used the ratio of Tregs: Th17 cells as an index, however, we did not observed remarkably difference on the ratio of Tregs to Th17 cells between NC and GC patients (6.41±2.46 *vs.* 6.19±1.40; Student’s *t* test, *P* = 0.728, Figure 3D).

### Blockade of Notch signaling did not affect the frequencies of Tregs and Th17 cells in GC patients

**Figure 4:**
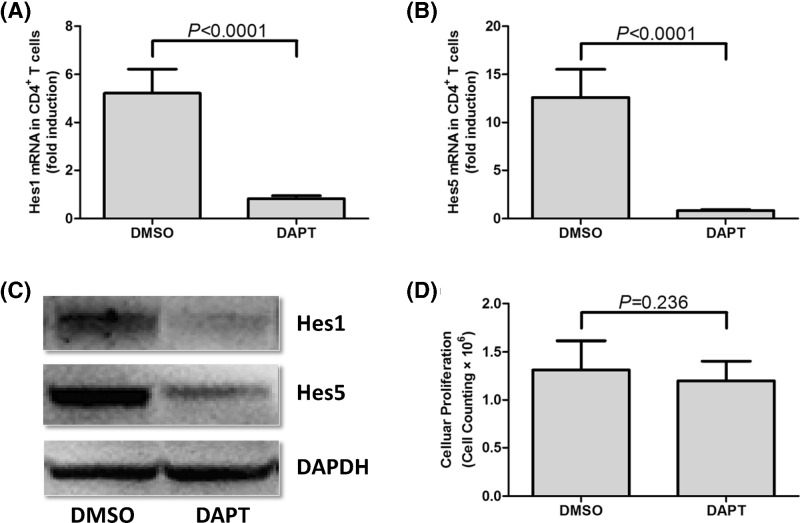
Expression of Notch signaling-related molecules (Hes1 and Hes5) and cellular proliferation of purified CD4^+^ T cells in response to DAPT treatment Purified CD4^+^ T cells from 15 GC patients were stimulated with DMSO or DAPT for 24 h. Both (**A**) Hes1 and (**B**) Hes5 mRNA levels were significantly down-regulated in response to DAPT stimulation. (**C**) Hes1 and Hes5 protein levels were also remarkably reduced in response to DAPT stimulation by Western blot tests. (**D**) Cellular proliferation was measured by cell counting kit-8, and there was no significant difference on cellular proliferation between DMSO and DAPT treatment. Columns presented as means, and bars presented as standard deviations. Paired *t* tests were used for comparisons.

**Figure 5:**
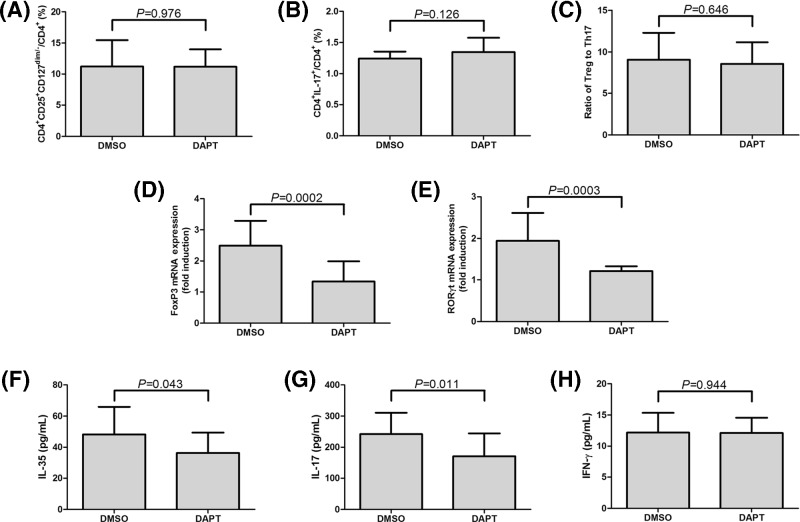
Influence of CD4^+^CD25^+^CD127^dim/−^ Tregs and Th17 cells in response to DAPT treatment in GC patients (*n* = 15) Purified CD4^+^ T cells were stimulated with DMSO or DAPT for 24 h. CD4^+^CD25^+^CD127^dim/−^ Tregs and Th17 cells was measured by flow cytometry. There were no remarkable differences on the percentages of (**A**) CD4^+^CD25^+^CD127^dim/−^ Tregs, (**B**) Th17 cells, and (**C**) ratio of Tregs: Th17 cells between DMSO and DAPT treatment. mRNA expression of FoxP3 and RORγt was measured by real-time PCR. (**D**) FoxP3 and (**E**) RORγt mRNA was significantly reduced in response to DAPT. IL-35, IL-17, and IFN-γ concentration was measured by ELISA. (**F**) IL-35 and (**G**) IL-17 expression was significantly reduced in response to DAPT. (**H**) There was no remarkable difference of IFN-γ production between DMSO and DAPT treatment. Columns presented as means, and bars presented as standard deviations. Paired *t* tests were used for comparisons.

DAPT, which is a γ-secretase inhibitor, was used to inactivate Notch signaling. Our previous study have been demonstrated that DAPT treatment down-regulated mRNA and protein expressions of Notch signaling-related molecules Hes1 and Hes5 [20]. CD4^+^ T cells from 15 GC patients were used in this experiment, and 10^6^ of CD4^+^ T cells were cultured in RPMI 1640 supplemented with 10% FBS and anti-CD3/CD28 in the presence of DMSO or DAPT for 24 h. DAPT stimulation significantly down-regulated Hes1 and Hes5 mRNA levels in cultured CD4^+^ T cells from GC patients (paired *t* tests, *P*<0.0001, Figure 4A,B). In accordance with reduced Hes1 and Hes5 mRNA, protein expressions of Hes1 and Hes5 also decreased in response to DAPT stimulation (Figure 4C). CCK-8 result revealed that there was no significant difference in absolute cell number between DMSO and DAPT treatment group (paired *t* test, *P* = 0.236, Figure 4D). DAPT treatment also did not affect the percentage of CD4^+^CD25^+^CD127^dim/−^ Tregs (11.21±4.26% *vs.* 11.17±2.82%; paired *t* test, *P* = 0.976, Figure 5A) or Th17 cells (1.24±0.11% *vs.* 1.35±0.23%; paired *t* test, *P* = 0.126, Figure 5B) in cultured CD4^+^ T cells. Thus, there was no remarkable difference on the ratio of Tregs to Th17 cells between cells with DMSO and DAPT treatment (9.07±3.22 *vs.* 8.57±2.62; paired *t* test, *P* = 0.646, Figure 5C). The expression of FoxP3 (transcriptional factor for Tregs) and RORγt (transcriptional factor for Th17 cells) was also measured in response to DAPT treatment. DAPT treatment significant down-regulated mRNA expression of both FoxP3 (paired *t* test, *P* = 0.0002, Figure 5D) and RORγt (paired *t* test, *P* = 0.0003, Figure 5E) in cultured CD4^+^ T cells. Similarly, both Tregs-secreting cytokine IL-35 (48.27±17.56 pg/ml *vs.* 36.20±13.25 pg/ml; paired *t* test, *P* = 0.043, Figure 5F) and Th17-secreting cytokine IL-17 (170.9±73.58 pg/ml *vs.* 242.0±68.70 pg/ml; paired *t* test, *P* = 0.011, Figure 5G) revealed notable reduction in response to DAPT stimulation in the supernatants of cultured CD4^+^ T cells. Moreover, there was no significant difference of IFN-γ production in cultured supernatants between DMSO and DPAT stimulation (12.18±3.18 pg/ml *vs.* 12.11±2.47 pg/ml; paired *t* test, *P* = 0.944, Figure 5H).

### Blockade of Notch signaling reduced the suppressive capacity of CD4^+^CD25^+^CD127^dim/−^ Tregs in GC patients

**Figure 6:**
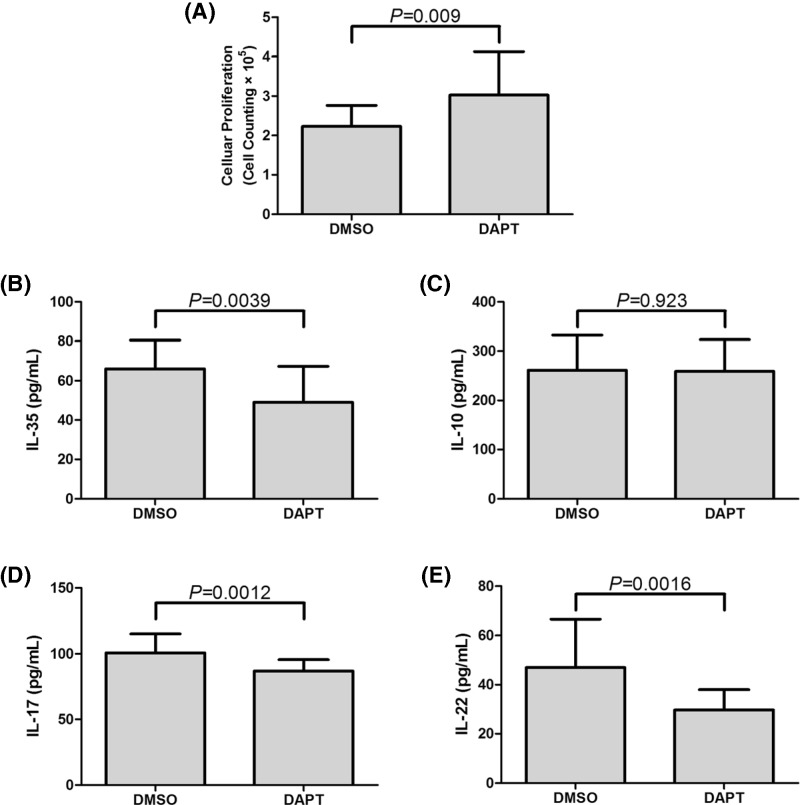
Inhibition of suppressive function of purified CD4^+^CD25^+^CD127^dim/−^ Tregs in response to DAPT stimulation in GC patients (*n* = 18) Purified CD4^+^CD25^+^CD127^dim/−^ Tregs from GC patients were stimulated with DMSO or DAPT for 24 h, and were co-cultured with CD4^+^CD25^−^ T cells at the ratio of 1: 4 for another 96 h. (**A**) Cellular proliferation of co-cultured cells were significantly increased in response to DAPT treatment. Levels of (**B**) IL-35, (**C**) IL-10, (**D**) IL-17, and (**E**) IL-22 in the supernatants of co-cultured cells were also measured by ELISA. DAPT notably reduced the levels of (**B**) IL-35, (**D**) IL-17, and (**E**) IL-22 in co-cultured cells in GC patients, however, (**C**) IL-10 expression did not show remarkably changes with DAPT treatment. Columns presented as means, and bars presented as standard deviations. Paired *t* tests were used for comparisons.

To investigate the direct role of Notch signaling in regulation of Tregs in GC patients, CD4^+^CD25^+^CD127^dim/−^ Tregs and CD4^+^CD25^−^ T cells were purified from eighteen GC patients. Tregs were treated with DAPT or DMSO for 24 h. 2.5×10^4^ of stimulated Tregs were co-cultured with autologous 10^5^ of CD4^+^CD25^−^ T cells in the presence of anti-CD3/CD28 for 96 h. Cells were harvested for proliferation assay, while supernatants were harvested for ELISA. The presence of DAPT notably inhibited the suppressive activity of Tregs, which presented as increased cellular proliferation in co-culture system ([3.03±1.09]×10^5^ vs. [2.23±0.54]×10^5^; paired *t* test, *P* = 0.009, Figure 6A). Furthermore, expressions of IL-35, IL-10, IL-17, and IL-22 in co-cultured supernatants were measured by ELISA. DAPT treatment significantly down-regulated IL-35 production in co-culture system (49.01±18.32 pg/ml vs. 66.06±14.47 pg/ml; paired *t* test, *P* = 0.0039, Figure 6B), however, there was no remarkable difference of IL-10 expression in the supernatants between DAPT and DMSO stimulation (259.3±63.94 pg/ml vs. 261.4±70.81 pg/ml; paired *t* test, *P* = 0.923, Figure 6C). Both IL-17 and IL-22 secretion was notably reduced in DAPT treated supernatants (IL-17: 86.95±8.59 pg/ml vs. 100.9±14.29 pg/ml; paired *t* test, *P* = 0.0012, Figure 6D; IL-22: 29.72±8.25 pg/ml vs. 46.94±19.59 pg/ml; paired *t* test, *P* = 0.0016, Figure 6E).

### Notch1 and Notch2 expression was reduced post proximal or total gastrectomy therapy

**Figure 7:**
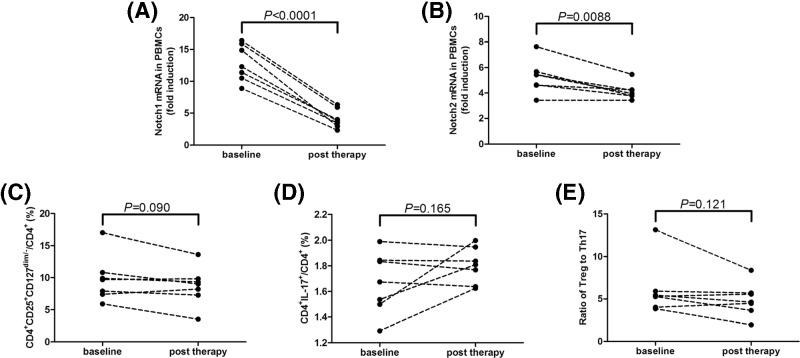
Notch1 and Notch2 mRNA expressions, CD4^+^CD25^+^CD127^dim/−^ Tregs and Th17 cells in response to proximal or total gastrectomy therapy in GC patients (*n* = 7) (**A**) Notch1 and (**B**) Notch2 mRNA expressions in PBMCs was notably down-regulated post operation. (**C**) CD4^+^CD25^+^CD127^dim/−^ Tregs and (**D**) Th17 cells percentage did not change significantly post operation. (**E**) The ratio of Tregs to Th17 was also comparable before and post operation. Individual level of each subject was shown. Paired *t* tests were used for comparisons.

Seven of all 51 GC patients received proximal or total gastrectomy therapy, and blood samples were collected 3–4 weeks post operation before chemotherapy. Notch1 and Notch2 mRNA expressions in PBMCs were notably down-regulated post operation (paired *t* tests, *P*<0.0001 and *P* = 0.0088, respectively, Figure 7A,B). However, both CD4^+^CD25^+^CD127^dim/−^ Tregs and Th17 cells percentage did not change significantly post operation (paired *t* tests, *P* = 0.090 and *P* = 0.165, respectively, Figure 7C,D). Thus, the ratio of Tregs to Th17 was also comparable before and post operation (paired *t* test, *P* = 0.121, Figure 7E).

## Discussion

In the present study, GC evolution induced elevations of Notch1 and Notch2 in both peripheral bloods and tumor tissues, indicating that Notch signaling might take part in GC progression. There were elevations of both CD4^+^CD25^+^CD127^dim/−^ Tregs and Th17 cells in GC patients. Moreover, purified CD4^+^ T cells could respond to γ-secretase inhibitor DAPT, leading to down-regulation of FoxP3/RORγt mRNA expression and reduced IL-35/IL-17 production without influencing the percentage of Tregs/Th17 cells. The suppressive function of Tregs was also remarkably inhibited in response to DAPT treatment. The current results indicated that Notch signaling pathway might regulate Tregs and Th17 cells activity in the pathogenesis of GC.

Notch signaling was one of the most commonly activated signaling pathways in cancers through ligand-dependent and ligand-independent mechanisms [25,26]. Notch signaling pathway amplification or activating mutations played vital roles in the progression and evolution of cancers, including leukemia [26], prostate cancer [27], ovarian cancer [28], colorectal carcinoma [29,30], and breast cancer [31], etc. Our current results indicated significant elevations of Notch1/2 mRNA in peripheral bloods and GC tissues, which were consistent with previous studies [17]. However, the role of these increased Notch signaling molecules in the pathogenesis of cancers still not completely understood. A set of Notch signaling pathway inhibitors has been developed based on the molecular structure of Notch receptor, ligands, and activators [32], as well as neutralizing antibodies, siRNA, shRNA, and miRNA targeting Notch pathways [33]. Administration of Notch inhibitors revealed antitumor activity in clinical and preclinical trials in various malignances, including thyroid cancer, non-small cell lung cancer, melanoma, sarcoma or desmoid tumors [34], and hepatocellular carcinoma [33]. In contrast, Notch signaling pathway inactivation was found to favor the process of epithelial–mesenchymal transition and promote bladder cancer progression, indicating the tumor suppressive factor of Notch in the bladder and urothelial cancer [35,36]. Previous studies suggested that Notch signaling inhibition attenuated GC stem cell traits and enhanced sensitivity of GC to chemotherapy [19,37]. Furthermore, we also found that gastrectomy therapy induced decreased expression of Notch1 and Notch2 in GC patients. Thus, we assumed that the elevation of Notch1/2 in GC patients might promote tumorigenesis in GC.

Notch ligands Delta-like 4 (DLL4) and Jagged-1 activated Notch signaling on CD4^+^CD25^−^ effector T cells, leading to significant elevation of their sensitivity to Tregs-mediated suppression, indicating an indirect modulatory capacity of Notch signaling pathway to Tregs [38]. Our previous study revealed that CD4^+^CD25^+^CD127^dim/−^ Tregs constitutively expressed Notch molecules with increased Notch1/2 mRNA expressions in response to chronic HCV infection [20]. Thus, Notch signaling pathway might direct regulate Tregs function. However, controversy remained as the modulatory function of Notch signaling pathway on Tregs. DLL1-mediated Notch signaling enhanced the conversion of human memory CD4^+^ T cells into FoxP3-expressiong Tregs [39]. Ting et al. also indicated that DLL4/Notch induced epigenetic regulation to maintain Tregs differentiation and function during pulmonary respiratory syncytial virus infection [40]. In contrast, Bassil et al. showed that DLL4-Notch signaling suppressed the pool of CD4^+^FoxP3^+^ Tregs in both periphery and central nervous system in experimental autoimmune encephalomyelitis (EAE) mouse model [41]. Rong et al. also demonstrated that Notch signaling pathway negatively regulated immunosuppressive activity of infiltrating Tregs in experimental autoimmune uveitis mouse model [42]. Our present study showed an elevation of CD4^+^CD25^+^CD127^dim/−^ Tregs percentage in the peripheral bloods of GC patients, which was consistent with previous results [9]. Similarly, accumulated CD4^+^CD25^+^FoxP3^+^ Tregs was also found in both peripheral bloods and tumor tissues in Epstein–Barr virus-associated GC [43]. Notch signaling inhibition reduced FoxP3 expression and IL-35 production in cultured CD4^+^ T cells from GC patients. More importantly, Notch inhibitor also directly inhibited the suppressive activity of purified CD4^+^CD25^+^CD127^dim/−^ Tregs, which presented as reduced cellular proliferation to effector T cells and less IL-35 secretion in co-cultured cells. The current data indicated that increased Notch receptors expression in GC patients might promote suppressive capacity of CD4^+^CD25^+^CD127^dim/−^ Tregs, leading to the immunotolerance and reduced tumor rejection.

Notch1 was activated in both mouse and human *in vitro*-polarized Th17 cells, and blockade of Notch signaling *in vivo* down-regulated Th17 differentiation, resulting in reduction in Th17-mediated disease progression in EAE [44], collagen-induced arthritis [45], and allergic asthma mouse models [46]. This regulatory activity controlled trafficking of IL-17 and metabolic regulators (including CD71 iron transporter and mTORC2 activity) within Th17 cells in a context-dependent manner [47]. In contrast, Jagged-1 activated Notch signaling suppressed cytokine-induced differentiation of Th17 cells via RORγt/IL-17A/IL-23 reduction [48,49]. Our current study also revealed an increased frequency of circulating Th17 cells in GC patients, which might contribute to GC development and metastasis [50]. In contrast with our current findings, Th17-secreting cytokine IL-17 was reduced in the serum from patients with gastrointestinal stromal tumors, neuroendocrine neoplasms, and lymphomas, but not in carcinoma [51]. This might be due to the different races of enrolled subjects because all the enrolled subjects are Asians. Moreover, blockade of Notch signaling decreased RORγt mRNA expression and IL-17 secretion in cultured CD4^+^ T cells from GC patients, indicating the maintenance of Th17 cells function by Notch signaling pathway in GC pathogenesis. Furthermore, inactivation of Notch signaling reversed Tregs/Th17 imbalance in patients with immune thrombocytopenia [52] and chronic hepatitis C [20]. Thus, elevation of Notch receptors might simultaneously regulate the functions of Tregs and Th17 cells in GC patients, although we did not find a significant imbalance between Tregs and Th17 cells. Interestingly, Notch signaling inhibition did not affect the percentages of Tregs and Th17 cells, however, reduced transcriptional factor mRNA expression and cytokines production. This indicated that DAPT stimulation might induce Tregs/Th17 cells exhaustion or dysfunction. Importantly, we found that GC resection only altered Notch receptors mRNA expression, but not affect Treg/Th17 balance, which was not consistent with the previous reports on either peripheral bloods [53] or tumor microenvironments [54]. This might be due to the relative short following-up time (only 3∼4 weeks post therapy) in our current study. The underlying mechanism related to the interaction between Notch and Th cells in regulation of tumorigenesis was needed to be further elucidated.

In conclusion, DAPT-induced inactivation of Notch signaling pathway did not affect Tregs-Th17 subset balance, however, induced the down-regulation of key transcriptional factors (FoxP3/RORγt). More importantly, Notch signaling inhibitor also dampened the suppressive activity of Tregs and cytokine production by Th17 cells in GC patients. The link between Notch signaling and Th cells might lead to a new therapeutic target for GC patients.

## Abbreviations

CCK-8: Cell Counting Kit-8
DLL4: Delta-like 4
DMSO: dimethyl sulfoxide
ELISA: enzyme-linked immunosorbent assay
FBS: fetal bovine serum
GC: gastric cancer
HCV: hepatitis C virus
IL: interleukin
NC: normal control
PBMC: peripheral blood mononuclear cell
RORγt: retinoic acid-related orphan receptor-γt
Th17: T helper 17
TNM: tumor–node–metastasis
Tregs: regulatory T cells
